# Human umbilical cord mesenchymal stem cell exosomes inhibit proliferation and promote apoptosis in an in vitro model of keloid

**DOI:** 10.1016/j.bbrep.2026.102727

**Published:** 2026-07-29

**Authors:** Wenjing Han, Jinjia Cui, Zijun Zhou, Huiru Zhuang, Hongmei Tang

**Affiliations:** Plastic and Cosmetic Surgery Department, Suzhou University Affiliated Second Hospital, 1055 Sanxiang Road, Suzhou, Jiangsu Province, 215000, China

**Keywords:** Keloid, Fibroblasts, Umbilical cord mesenchymal stem cell, Exosomes

## Abstract

**Background:**

Keloid is a pathologic scar formation resulting from trauma, burns, surgery, or unknown causes, leading to deformity and varying degrees of functional impairment, significantly impacting the physical and mental well-being as well as the quality of life of affected individuals. Studies have indicated that Mesenchymal Stem Cells (MSCs) can effectively promote wound healing in various injuries, reducing the formation of keloid. This study aims to explore an in vitro model of keloid and investigates the effects of exosomes from human Umbilical Cord MSCs (HUCMSC) on fibroblast proliferation and apoptosis in keloid model.

**Methods:**

Methods include isolating primary fibroblasts, extracting HUCMSC exosomes (HUCMSC-Exos), and establishing a histamine-induced keloid model in vitro. Results show dose-dependent inhibition of keloid fibroblast activity by HUCMSC-Exos, affecting proliferation, apoptosis, and cell cycle. Through gene sequencing analysis, we investigated the abnormal gene expression and signaling pathway changes of histamine-induced keloid fibroblast before and after co-cultivation with HUCMSC-Exos.

**Results:**

It turns out that HUCMSCs-Exos effectively inhibit the proliferation of keloid fibroblasts, promoting apoptosis and exhibiting a dose-dependent effect. We also proved that HUCMSCs-Exos regulated genes and inhibit signaling pathway by upregulating the expression of intracellular genes, ultimately inhibiting keloid fibroblasts proliferation activity and enhancing apoptosis. The research found that HUCMSC-Exos as potential regulators of keloid fibroblast behavior, offering insights for keloid treatment strategies.

## Highlights

• Histamine used for first time to induce keloid fibroblast overgrowth.
• First use of HUCMSC-Exos to treat keloid in vitro model.
• Study used primary cells from real patients in a clinical setting.
• HUCMSC-Exos show low immunogenicity and are easily accessible.
• Exosomes inhibit fibroblast growth and promote apoptosis in keloid.

## 1 Introduction

Keloid disease, a pathologic scar formation resulting from trauma, burns, surgery, or unknown causes, is characterized by a tendency for recurrence after treatment, leading to deformity and varying degrees of functional impairment [1]. This recurrence significantly impacts the physical and mental well-being as well as the quality of life of affected individuals [2]. As a common condition in plastic surgery, keloid poses a significant challenge to the medical field today [3].

Abnormal proliferation of fibroblasts is a major contributor to excessive and persistent growth in keloid, leading to over production of collagen and cytokines [4,5]. Given the current lack of ideal therapeutic methods for pathologic scars in clinical practice, comprehensive treatment remains the primary approach. Some researchers have suggested that the unsatisfactory treatment outcomes in keloid may be correlated with the deficiency in apoptosis of fibroblasts [6,7]. Fibroblasts proliferate and gradually differentiate into myofibroblasts with enhanced secretion functions during the proliferation stage of wound healing. Myofibroblasts can secrete α-SMA, which has a promoting effect on wound healing. It also secretes COL-Ⅰand COL-Ⅲ and has a positive influence on completing extracellular matrix (ECM) remodeling [8]. Investigating the mechanisms of fibroblast apoptosis deficiency in scar tissue and effectively inducing apoptosis in fibroblasts associated with pathologic scars is of significant importance for the clinical treatment of keloid [9,10].

Studies have indicated that Mesenchymal Stem Cells (MSCs) can effectively promote wound healing in various injuries, reducing the likelihood of pathologic scar formation [11,12]. Human Umbilical Cord Mesenchymal Stem Cells (HUCMSCs) are currently a popular focus in research on skin wound repair [13,14]. Despite the potential of MSCs to promote wound healing and prevent keloid formation [15], limitations such as difficulties in preservation, immune rejection, and the risk of mutation exist in the application of stem cells [16]. Therefore, there is a need for an alternative tissue with the therapeutic effects of MSCs while overcoming their limitations.

Extracellular vesicles, specifically exosomes, secreted by MSCs, participate in various physiological activities and provide an ideal therapeutic means for achieving cell-free wound healing [17,18] In the context of keloid pathophysiology, MSC-derived exosomes have been reported to exert anti-fibrotic effects through multiple converging mechanisms: (1) inhibiting fibroblast hyperproliferation by transferring regulatory miRNAs that target pro-growth signaling cascades [19,20]; (2) suppressing excessive collagen synthesis through downregulation of the TGF-β/Smad pathway and reduction of COL-I and COL-III deposition [20,21]; (3) restoring the apoptotic balance in keloid-associated fibroblasts that is characteristically deficient in pathological scarring [21]; and (4) modulating the local pro-inflammatory microenvironment by attenuating NF-κB signaling and reducing pro-inflammatory cytokine production [19]. Recent advances have also introduced engineered exosomes — surface-modified or loaded with specific therapeutic cargo — as next-generation platforms to amplify anti-fibrotic effects with greater precision [22]. The understanding of the regulatory mechanisms and components of HUCMSCs-Exos in the pathological development of keloid is still in its early stages and requires further in-depth investigation [1]. This study aims to explore the impact of HUCMSCs-Exos on the proliferation and apoptosis of keloid fibroblasts, establish a model of keloid in vitro, and investigate the molecular mechanisms underlying the regulation of keloid fibroblast proliferation and apoptosis by HUCMSCs-Exos.

## 2 Materials & methods

### 2.1 Cell isolation and culture

#### 2.1.1 Keloid fibroblasts

Keloid fibroblasts were obtained from 8 patients with keloid in the Department of Plastic Surgery at the Second Affiliated Hospital of Suzhou University. This study and the research including the experimental procedures were approved by the Second Affiliated Hospital of Soochow University (EC2023-154). Inclusion criteria: (1) Meets clinical keloid diagnostic criteria [23]; (2) The scar tissue sampling site has no ulcers, infections, and has not received any radiation, drug injection, or topical medication treatment. Exclusion criteria: The experimental subject has infections, malignant tumors, and other organic lesions in other parts of the body. The patients had signed the informed consent. The primary fibroblasts derived from three different patients were named as FC601590, FC601593, FC601596. The excised keloid tissue was removed from the epidermis and delivered to the laboratory within 1 h. In a sterile environment, it was repeatedly rinsed with normal saline, soaked in 0.05% chlorhexidine for 5-10 min, trimmed into 1 cm^3^ small pieces, washed with PBS, then added about 10 times the amount of collagenase solution (concentration: 0.25 g/L, prepared with DMEM medium), digested in a 37°C thermostatic water bath with shaking for 2 h, filtered with a screen (100 mesh) to remove gelatinous protein and undigested tissue. The remaining filtrate was centrifuged (1500 r/min, 5 min), the supernatant was discarded and an appropriate amount of DMEM medium (DMEM: FBS: double antibody = 500:50:5) was added. Then it was blown and mixed well, then a sample was taken for counting. The cell concentration was adjusted to 2×104/mL and 10 mL of cell suspension was added to a 90 mm Petri dish. The cells were cultured in a 37°C cell culture incubator with 5% CO_2_. The cell culture medium was changed every 2 days. When the cells grew (80% to 90%), it was digested with 0.25% trypsin and the cell was passaged in the way of 1:4. In the experiment, 1st to 2nd generation keloid fibroblast was used.

#### 2.1.2 Human umbilical cord mesenchymal stem cell(HUCMSCs)

HUCMSCs were obtained from the umbilical cord of three fetuses delivered at the Obstetrics and Gynecology Department of the Second Affiliated Hospital of Suzhou University. Inclusion criteria: Umbilical cord tissue belonging to normal delivery fetuses. Exclusion criteria: (1) There are related complications or underlying diseases during pregnancy; (2) The fetus has hereditary diseases. The mothers had signed the informed consent. The adventitia of umbilical cord from healthy donors was removed, blood vessels were removed, and umbilical cord was trimmed into 2-3 cm segments. After being sheared, it was placed in a sterile centrifuge tube, and α-MEM medium was added to 15 mL. Then added CollagenaseⅠwith the same volume, and digested it overnight in a 5% CO_2_ incubator at 37°C. The next day, trypsin was added to make the final concentration of 0.05% and digested for 1-2 h. The digestion was terminated by adding fetal bovine serum. After collecting the digestive solution, PBS was added to dilute 10 times the volume and filtered through a 200 mesh filter screen. Centrifuged for 20 min at 2500 r/min, washed the precipitate with PBS twice, and centrifuged for 10min at 1500r/min each time. After cell counting, the cells were seeded into Petri dishes and cultured in a 5% CO_2_ incubator at 37°C.

### 2.2 The histamine-induced keloid model in vitro

The number of cells in the cell suspension was determined by cell counting plate, and cell suspensions with different histamine concentrations were prepared for 5 groups. To establish the histamine-induced keloid model in vitro, primary keloid fibroblasts (passage 1–2, isolated as described in Section 2.1.1) were used. ^−3^ mol/L, 1.0 × 10^−4^ mol/L, 1.0 × 10^−5^ mol/L, 1.0 × 10^−6^ mol/L). 100 μL cell suspension of each group were inoculated with cells (5 multiple wells in each group), and cultured in a 37°C cell incubator with 5% CO_2_ for 2 h. After the cells were adherent, CCK-8 reagent 10 μL was added to each well. The incubation was continued in the incubator for 2 h.

### 2.3 Extraction and isolation of HUCMSCs-Exos

The supernatant of cell culture was centrifuged at 3000 g for 20 min, and the supernatant was taken. The ultra pure chromatographic column was washed with cold PBS filtered by the 0.22 μm filter. The supernatant sample after centrifugation was taken into the chromatographic column. Effluent was collected by a clean 2 mL centrifuge tube, which was the HUCMSCs-Exos solution. HUCMSCs were maintained in standard complete medium (α-MEM supplemented with 10% FBS and 1% penicillin/streptomycin) throughout the experiment. No serum starvation protocol was employed prior to supernatant collection, in order to preserve the native biological composition of the secreted vesicles under physiological culture conditions. Supernatant was collected from sub-confluent cultures (70–80% confluency) at 48-h intervals.

### 2.4 Identification and purification of HUCMSCs-Exos

#### 2.4.1 Transmission electron microscopy

5 μL of HUCMSCs-Exos solution was added to Formvar carbon sample loading copper grid, and 100 μL of PBS solution was added to the sealing film for cleaning. At first, the copper mesh was dropped on 50 μL of glutaraldehyde solution (1%) for 5 min, cleaned with ddH_2_O for 2 min, and repeated for 8 times, and dropped on 50 μl of pH7.0 uranyl oxalate for 5 min.Then it was placed at 50 μL methylcellulose droplet for 10 min (operation on ice), put on the stainless steel ring, absorbed the excess liquid with filter paper and dried in air for 5-10 min. Finally, the copper mesh was put in the sample box and taken electron microscope photos at 80 kV.

#### 2.4.2 Nanoparticles tracking

The sample cell was cleaned with deionized water, instrument was calibrated with the polystyrene microspheres and sample pool was washed with 1× PBS. The HUCMSCs-Exos solution was diluted with 1× PBS for the machine detection.

#### 2.4.3 Western-blotting

4% concentrated and 10% separation gel were prepared. 10 μL of HUCMSCs culture supernatant and 10 μL of HUCMSCs-Exos solution were taken and added to the lane. The voltage was 60 V and concentrated for 30 min. Voltage was 120 V and separated for 60 min. The current was 200 mA and electric transfer was continued for 1h. 5% milk in 1× TBS-T buffer was blocked for 1 h. Primary antibodies were incubated and incubated 4°C overnight. The membrane was washed with 1× TBS-T buffer for three times. Secondary antibodies were incubated in the shaker for 1h at room temperature. The membrane was washed with 1× TBS-T buffer for three times again. The image was developed using hypersensitive ECL chemical light kit. Gray value was analyzed by Image J software.

### 2.5 Cell proliferation activity assay

CCK-8 colorimetric assay was used to measure Cell proliferation activity assay. 2.0 × 10^4^/mL keloid fibroblast in logarithmic growth phase were seeded in 96-well cell culture plates, 100 μL per well, with different HUCMSCs-Exos culture medium and different time points for intervention. According to the instructions of the CCK-8 kit, 10 μL of CCK-8 reagent was added to each well, and then moved to the incubator for 120 min of incubation, during which the conditions were set at 37°C temperature, 5%CO_2._ With the help of the enzyme-linked immunodetector, the number of living cells was indirectly reflected by the OD_450nm_ absorption value. Then, proliferation inhibition (IR) was found. lR = [(average of OD control well - average of OD delivery well)/average of OD control well] 100%.

### 2.6 Apoptosis level assay

AnnexinV-positive flow cytometry was used to measure apoptosis level assay. The cell concentration was adjusted to 2.0 × 10^4^/mL, added to 24-well plates, given different group treatments. Followed by trypsin digestion and PBS washing twice, the cell concentration was adjusted to (1∼5)× 10^5^/well. After cell suspension with 100 μL of 1×binding buffer, 5 μL of Annexin V FITC, 60 μl of 1 Annexin V Binding Buffer were added for 10 min, followed by 5 μL of PI reagent, 120 μL of 1×Annexin V Binding Buffer. The samples were mixed and tested by flow cytometry (BD Company, America) to analyze the percentage of Annexin V positive and PI negative cells with built-in analysis software.

### 2.7 Cell cycle change assay

Special kit for cell cycle detection was used to measure cell cycle change assay. The cell concentration was adjusted to 2.0 × 10^4^/mL, then added to 24-well plates for different group treatments. With trypsin digestion, the cells were collected after washing twice in PBS. According to the kit instructions, cells were fixed with 70% ethanol for 4°C overnight. After PBS washing in the next day, 20 μL of RNase A was added and incubated at 37°C for 30 min. With the way of light avoidance operation, Pl dye solution was added, bathed by ice for 30 min, and filtered by 300 mesh screen after staining. The cell concentration was adjusted to l.0 × 10^9^/L. The specimens were examined by flow cytometry and analyzed with their own analysis software to record the percentage of cells in G0/G1, S, and G2/M phases in each group.

### 2.8 Target gene screening

Three samples from histamine-induced keloid fibroblasts after co-cultivated with HUCMSCs-Exos group and three samples from histamine-induced keloid fibroblasts with non intervention group were sent to Lianchuan Biotechnology for transcriptome sequencing. The sequencing data obtained was compared with the reference genome of the species using HISAT2 (https://daehwankimlab.github.io/hisat2/version:hisat2-2.0.4). Differential gene expression analysis between the two groups was conducted using DESeq2 software. The screening criteria applied were a significance level of P < 0.01 and | log2 fold change | > 1. At the end, the differentially expressed genes were imported into R software and ggplot2 was used to generate both the volcano plot and the heat map.

### 2.9 Molecular screening of signaling pathway

Functional enrichment analysis was conducted on the differentially expressed gene data, using GSEA (v4.1.0) and MSigDB software. The analysis incorporated Gene Ontology (GO) and Pathway Analysis (PA). GO analysis includes the three categories: molecular function, biological processes and cellular components. For pathway analysis, two databases, Database for Annotation, Visualization and Integrated Discovery (DAVID) and Ingenuity Pathway Analysis (IPA), were employed. The DAVID database specifically analyzed signal pathways related to the Kyoto Encyclopedia of Genes and Genomes (KEGG) database [24].

### 2.10 Statistical analysis

The software SPSS22.0 was used in data processing and analysis process. The measurement value were presented in the form of ($\bar{x}\pm s$), with analysis of variance and one-way ANOVA, setting the test level α = 0.05. P < 0.05 was considered to indicate a statistically significant difference.

## 3 Results

### 3.1 Morphology of keloid fibroblast

The boundary of Keloid fibroblasts was relatively clear, and the cell body was large, showing a rhombic or irregular elongated type. The cell protruded outward with 2-3 protrusions of different lengths, and the nucleus was quasi circular or long oval (Fig. 1A). With the increase of culture days, the cells were arranged closely, the gaps became smaller and smaller, and the cells were parallel to each other. After 1 week of culture, the cells showed a radial and vortex regular arrangement (Fig. 1B), and the fusion degree reached 80%∼90%, which met the needs of subsequent experiments.

**Fig. 1:**
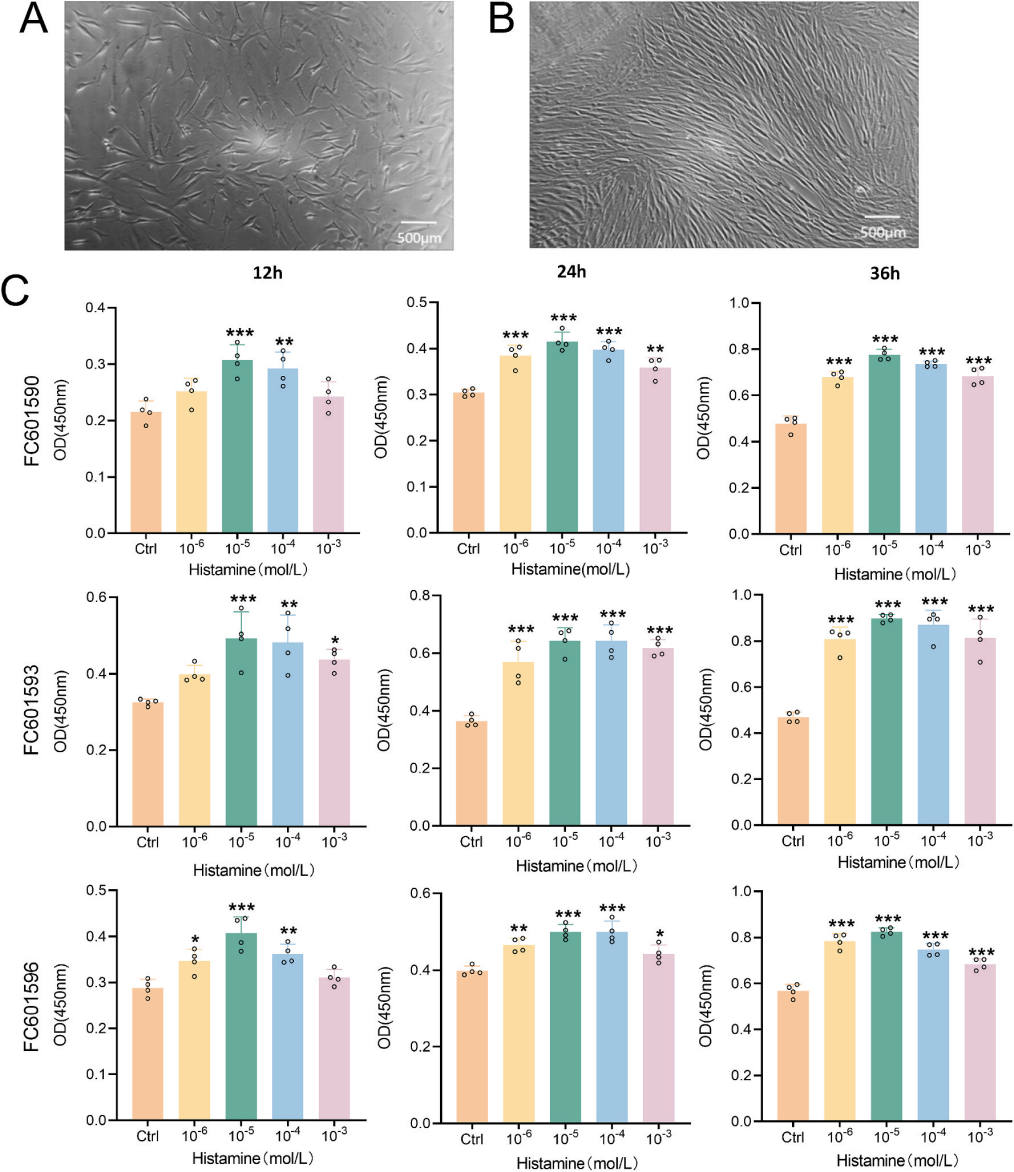
Keloid fibroblast morphology and histamine-induced keloid model in vitro. (A) Morphology of primary keloid fibroblasts at early passage, showing rhombic or irregular elongated cell bodies with 2–3 protrusions and quasi-circular or oval nuclei. (B) After 1 week of culture, cells exhibited a radial and vortex-like arrangement with 80–90% confluency. (C) CCK-8 proliferation assay of keloid fibroblasts treated with different concentrations of histamine (0, 10^−6^, 10^−5^, 10^−4^, 10^−3^ mol/L) at 12 h, 24 h, and 36 h time points. The 10^−5^ mol/L histamine concentration with 36 h treatment showed the most significant proliferation (P < 0.001).

### 3.2 Histamine induced keloid model in vitro

To establish a histamine induced keloid model in vitro, specific histamine induced concentrations involved were 0, 10^−6^ mol/L, 10^−5^ mol/L, 10^−4^ mol/L, 10^−3^ mol/L, with time points of 12h, 24h and 36h. The control group was primary fibroblast culture. The results (Fig. 1C) showed that the proliferation rate of the cells with different histamine concentrations was increased compared to the control group, with increasing time. The appropriate amount of histamine concentration could promote the proliferation of keloid fibroblast. Compared with each concentration, the 10^−5^ mol/L concentration promoted better, and the proliferation effect was more pronounced with the treatment time of 36h. Statistically significant difference (P < 0.001). In subsequent studies, we selected the concentration of histamine-induced keloid fibroblast as 10^−5^mol/L. Keloid in vitro referred to being under the treatment of histamine and keloid fibroblasts referred to fibroblasts treated with histamine.It is noteworthy that histamine exerts its proliferative effects primarily through activation of H1/H2 receptor-coupled second-messenger cascades (cAMP, IP_3_/Ca^2+^) that accelerate cell cycle entry, rather than inducing overt morphological changes detectable by standard brightfield microscopy. Therefore, the CCK-8 quantitative proliferation assay (Fig. 1C) constitutes a rigorous and appropriate functional readout for validating the histamine-induced keloid model, which was designed to recapitulate the hyper-proliferative phenotype characteristic of keloid in vivo.

### 3.3 Characterization of HUCMSCs-Exos

Transmission electron microscopy (TEM), Western Blotting (WB) and nanoparticles tracking were used to identify particles from HUCMSCs. As shown in Fig. 2A, the particles were in a tiny membrane vesicular shape under TEM. Because of its small volume and the structure of the lipid bilayer, it can easily and freely move between cells and convey biological signals. WB was used to identify the HUCMSCs-Exos-specific phenotypic markers CD9, CD63, and CD81 on the microparticle surface (Fig. 2B). The particles had a diameter of about 50-200 nm and had a high purity under nanoparticles tracking (Fig. 2C).

**Fig. 2:**
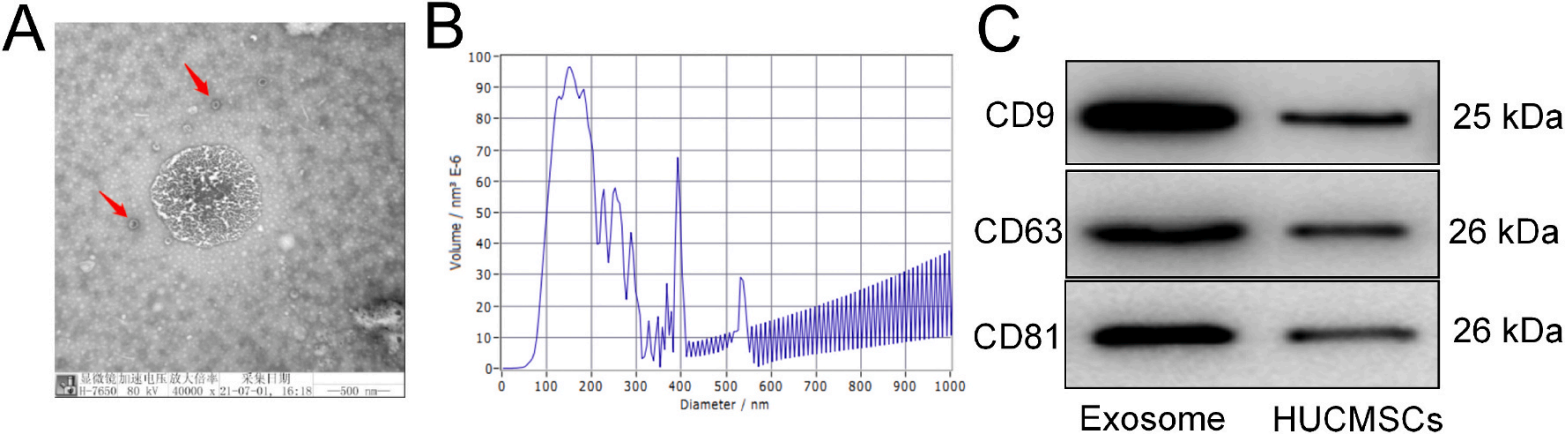
Characterization of HUCMSCs-derived exosomes. (A) Transmission electron microscopy (TEM) image of HUCMSCs-Exos showing typical membrane vesicular morphology. (B) Western blotting results confirming expression of exosomal markers CD9, CD63, and CD81. (C) Nanoparticle tracking analysis showing HUCMSCs-Exos with a diameter of approximately 50–200 nm and high purity.

### 3.4 HUCMSCs-Exos inhibited the proliferation of keloid fibroblast

Five different HUCMSCs-Exos concentrations involved were 0, 5 μg/mL, 10 μg/mL, 20 μg/mL, and 40 μg/mL. The control group wasn't added anything other than basic culture conditions. The vehicle group was induced by histamine and HUCMSCs-Exos concentrations was 0. The HUCMSCs-Exos concentrations of the other four histamine-induced groups were 5 μg/mL, 10 μg/mL, 20 μg/mL, and 40 μg/mL. All groups were taken three samples and were detected cell viability at time points of 1d, 2d, and 3d. The results (Fig. 3) showed that the proliferation rate of human keloid fibroblast in each group was different. The results showed that the proliferation rate of HUCMSCs-Exos decreased compared with the control group, with the highest proliferation inhibition of keloid fibroblast at 40 μg/mL. The difference was statistically significant (P < 0.001). In conclusion, the HUCMSCs-Exos reversed the trend of histamine-induced proliferation in keloid fibroblast and reduced the proliferative activity of keloid fibroblast.

**Fig. 3:**
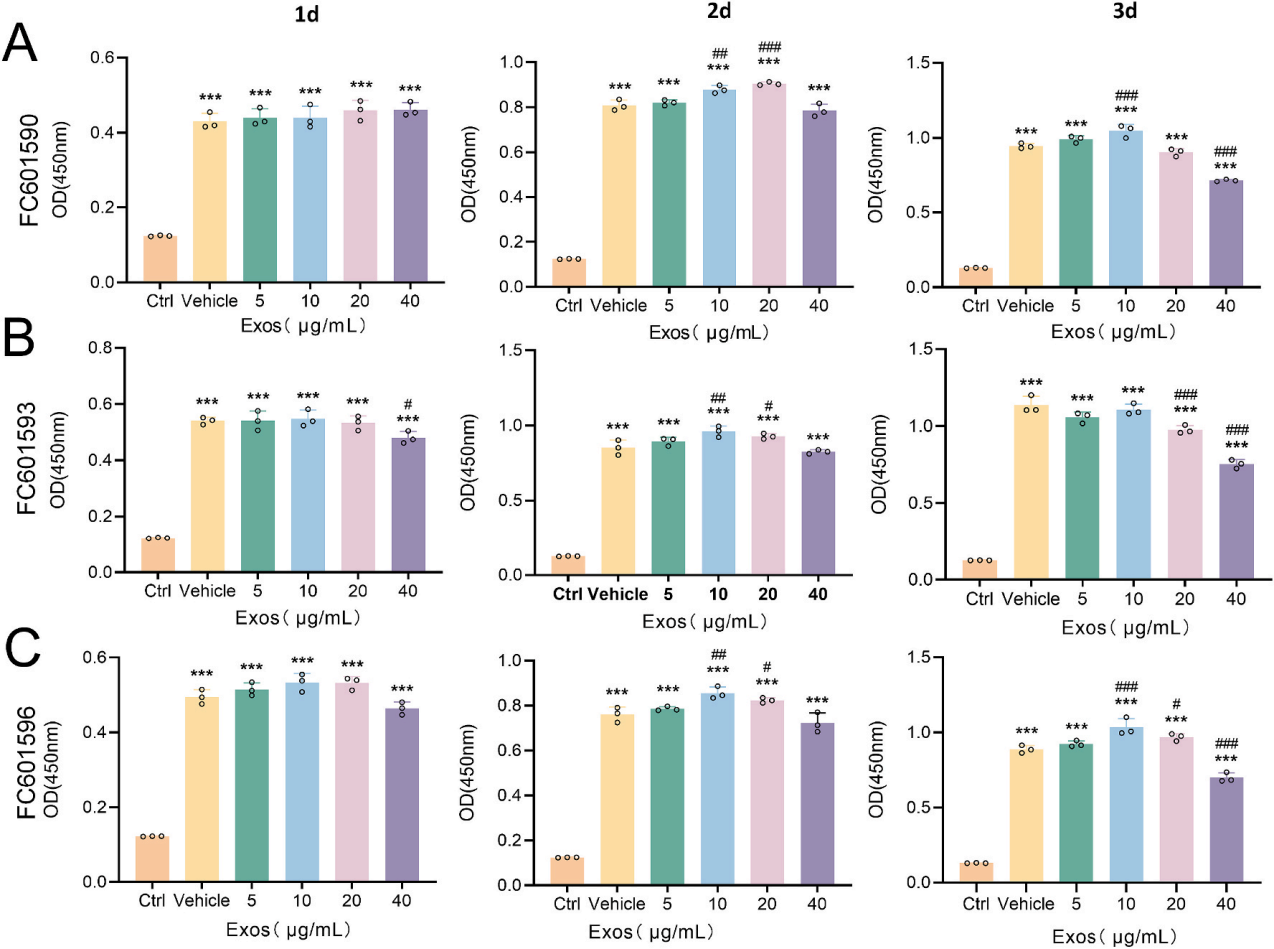
HUCMSCs-Exos inhibit keloid fibroblast proliferation in a dose-dependent manner. CCK-8 proliferation assay results of histamine-induced keloid fibroblasts co-cultured with HUCMSCs-Exos at concentrations of 0, 5, 10, 20, and 40 μg/mL at 1 d, 2 d, and 3 d. The 40 μg/mL HUCMSCs-Exos concentration showed the highest inhibition of keloid fibroblast proliferation (P < 0.001).

### 3.5 HUCMSCs-Exos promoted the apoptosis level of keloid fibroblast

40 μg/mL of HUCMSCs-Exos concentration with the highest proliferation inhibition was chosen to co-cultivate with keloid fibroblast and histamine to detect the apoptosis level. The apoptosis level was compared at three time points involved 1d, 2d, 3d of keloid fibroblast. The results (Fig. 4A) showed that the apoptotic capacity of keloid fibroblast in each group showed variability. With the extension of time, the apoptosis ability of keloid fibroblast in each group showed an upward trend after co-cultivation with HUCMSCs-Exos. The difference was statistically significant (P < 0.05).

**Fig. 4:**
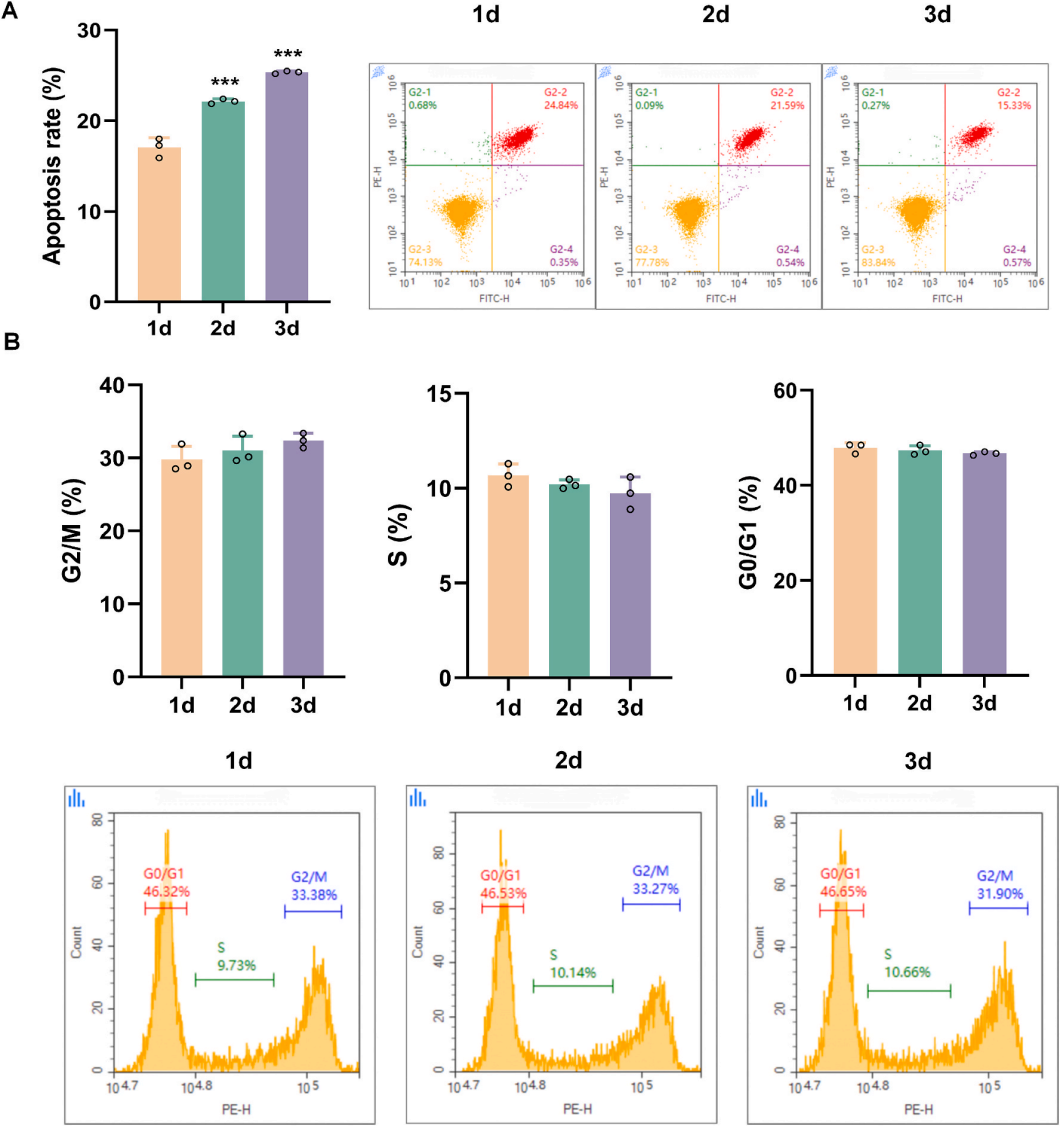
HUCMSCs-Exos promote apoptosis and alter cell cycle distribution in keloid fibroblasts. (A) Flow cytometry results (Annexin V-FITC/PI staining) showing the apoptosis rate of histamine-induced keloid fibroblasts co-cultured with 40 μg/mL HUCMSCs-Exos at 1 d, 2 d, and 3 d (P < 0.05). (B) Cell cycle analysis by flow cytometry (PI staining) showing the proportion of cells in G0/G1, S, and G2/M phases. HUCMSCs-Exos treatment led to a significant decrease in S-phase proportion, indicating reduced proliferative activity.

### 3.6 HUCMSCs-Exos altered the cell cycle ratio of keloid fibroblast

The treatment of keloid fibroblast was as same as 3.5. The cell cycle ratio was compared at three time points involved 1d, 2d, 3d of keloid fibroblast. The cell cycle was mainly divided into G1 phase, S phase and G2 phase. G1 phase was a period from mitosis to pre DNA replication, which was called pre synthesis. During this period, substance metabolism was more active, RNA and protein were rapidly synthesized, and the cell volume increased significantly. S phase was the key period in the cell cycle, which was the DNA synthesis phase, during which enzymes required for DNA replication are synthesized. G2 phase was the late stage of DNA synthesis, which was the preparation phase of mitosis. During this period, DNA synthesis was terminated. When the concentration of HUCMSCs-Exos intervention was 40 μg/mL, the mitotic activity of histamine-induced keloid fibroblast gradually decreased with increasing time, and the proportion of S phase was significantly down-regulated, suggesting a gradual decrease of keloid fibroblast (Fig. 4B). It shows that HUCMSCs-Exos could reverse the trend of histamine-induced proliferation rate of keloid fibroblast, which was decreasing the proliferative activity of keloid fibroblast. In G2/M phase, the mean of 1d, 2d, 3d is 0.2977, 0.3102 and 0.3238, the variance is 2.68 × 10^−3^; In S phase, the mean of 1d, 2d, 3d is 0.1068, 0.3102 and 0.3238, the variance is 3.624 × 10^−4^; In G0/G1 phase, the mean of 1d, 2d, 3d is 0.4788, 0.4736 and 0.4671, the variance is 6.608 × 10^−4^.

### 3.7 Gene sequencing data analysis results

**Fig. 5:**
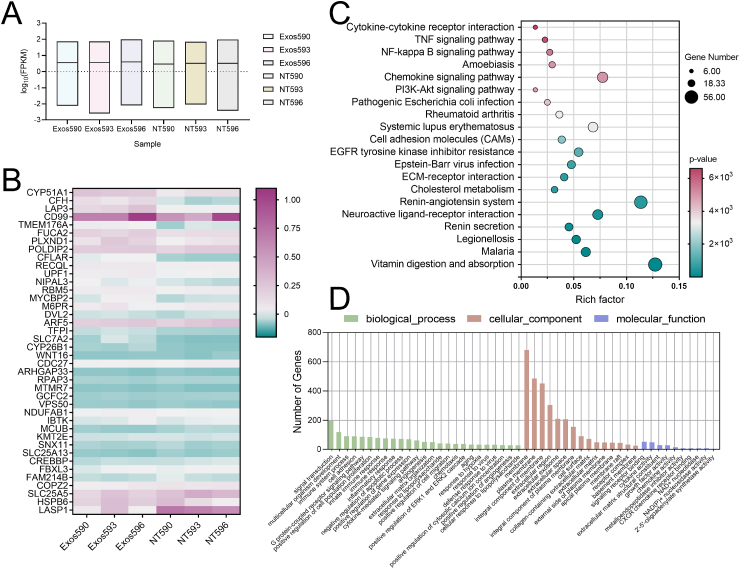
Transcriptomic analysis of keloid fibroblasts co-cultured with HUCMSCs-Exos. (A) Box plot of normalized gene expression levels across all samples, showing consistent medians indicating data comparability. (B) Heat map of differentially expressed genes, highlighting upregulated genes (CHRM2, KLF2, PSG1, KRT7) in keloid cells after HUCMSCs-Exos co-cultivation. (C) KEGG pathway enrichment bubble plot showing the top 20 enriched pathways, including Cytokine-Cytokine Receptor Interaction, TNF signaling pathway, and NF-κB signaling pathway. (D) Gene Ontology (GO) enrichment analysis covering biological processes, cellular components, and molecular functions of differentially expressed genes.

Analysis of the sequencing data revealed that keloid fibroblast co-cultured with HUCMSCs-Exos expressed 1105 up-regulated genes and 728 down-regulated genes. After gene-normalization (Fig. 5A), the data demonstrated a consistent median of gene expression intensity among gene chips, indicating the comparability of sequencing data.

### 3.8 Visualization of differentially expressed genes

The differentially expressed genes were imported into R software to generate the heat map (Fig. 5B) for visualization. In the heat map, the horizontal axis represents the gene sample, while the vertical axis represents the differentially expressed genes after screening; Different colors were used to represent differences in gene expression. If the color changes from blue to red, it indicates a shift from low to high expression levels. Blue and red respectively represented low and high expression genes. Upon analysis, it was found that the related genes CHRM2, KLF2, PSG1, KRT7 were all highly expressed in keloid cell samples after HUCMSCs-Exos co-cultivation. The high expression levels of those genes in the cell samples suggest a potential regulatory role in the pathological development of keloid.

### 3.9 GO and KEGG enrichment analysis

The differentially expressed genes mentioned above were subjected to GO and KEGG enrichment analysis. GO analysis covered the biological processes, cellular component, and molecular function of the genes (Fig. 5D). For KEGG analysis, the 20 KEGG pathways with the lowest p-value were selected to generate the bubble plot (Fig. 5C). The vertical axis represents the KEGG pathway, while the horizontal axis represents enrichment, reflecting the proportion of differentially expressed genes in the relevant KEGG signaling pathways. The color closeness to red signifies higher significance in enrichment, and the bubble size indicates the number of differentially expressed genes in the pathway. From the above data, the pathways with a higher number of enriched genes include the Cytokine Receptor Interaction pathway, TNF signaling pathway, NF-kappa B signaling pathway, Amoebias pathway, Chemokine signaling pathway, P13k-Akt signaling pathway.

## 4 Discussion

Histamine can effectively promote the growth and proliferation of fibroblasts and enhance the expression of collagen [25]. Therefore, at the beginning of the experiment, we treated keloid fibroblast with histamine to establish a keloid model in vitro. During the experiment, we analyzed the morphology of keloid fibroblast. The results showed that keloid fibroblast had clear boundaries, larger cell bodies, a rhomboid or irregular elongated shape, and 2-3 different lengths of protrusions extending outward. The cell nuclei were generally round or elongated, and after 1 week of cultivation, the cells exhibited a radial and vortex-like regular arrangement with a fusion rate of 80%∼90%, meeting the requirements for subsequent experiments. In the histamine induction process, concentrations of 0, 10^−3^mol/L, 10^−4^mol/L, 10^−5^mol/L, 10^−6^mol/L were used, and time points were set at 12h, 24h, and 36h. Compared to the control group, the proliferation rates of cells in all histamine groups showed an increasing trend. With prolonged time, the proliferation rates significantly increased. Among them, the concentration of histamine at 10^−5^mol/L, treated for 36h, showed the most significant proliferation effect, with statistically significant differences. This concentration was chosen for subsequent studies. Overall, histamine induction in keloid fibroblast suggests a successful modeling, showing differences from normal skin fibroblast. The optimal histamine intervention concentration for keloid fibroblast was determined to be 10^−5^mol/L, providing a foundation for further research.

After that, we co-cultured keloid fibroblast induced by histamine with HUCMSCs-Exos, attempting to analyze the impact of HUCMSCs-Exos co-cultivation on keloid fibroblast proliferation and apoptosis. The purity of extracted HUCMSCs-Exos was analyzed first, showing a relatively single peak in the process of extracting exosomes from HUCMSCs supernatant, indicating high extraction purity. HUCMSCs-Exos intervention concentrations were 0, 5 μg/mL, 10 μg/mL, 20 μg/mL, 40 μg/mL, and time points were set at 1d, 2d, and 3d. The results indicated that, compared to the control group, the proliferation rates of cells in all HUCMSCs-Exos co-cultured groups showed a decreasing trend. The inhibition rate of cell proliferation was most pronounced at an HUCMSCs-Exos concentration of 40 μg/mL, exhibiting a clear dose-dependent relationship with statistically significant differences. In conclusion, HUCMSCs-Exos can reverse the rising trend of histamine-induced keloid fibroblast proliferation and reduce their proliferative activity. Flow cytometry was then used to analyze the proportion of keloid fibroblast in different stages of cell division, including G1 phase, S phase, and G2 phase. As the concentration of HUCMSCs-Exos increased to 40 μg/mL, the mitotic activity of histamine-induced keloid fibroblast gradually decreased over time, with a significant decrease in the proportion of cells in the S phase and a decline in cell activity, showing statistically significant differences. This suggests that HUCMSCs-Exos can reverse the trend of increased proliferation rate induced by histamine in keloid fibroblast and reduce their proliferative activity. The results of this study show that HUCMSCs-Exos co-cultivation significantly promotes the trend of apoptosis in histamine-induced keloid fibroblast. It is hoped that through HUCMSCs-Exos, the pathological development of keloid can be inhibited, providing valuable insights for clinical research.

We acknowledge certain limitations of the present study. The transcriptomic analysis, while controlled and rigorous, represents a hypothesis-generating investigation into the mechanistic basis of HUCMSCs-Exos effects; protein-level validation of the identified target genes (PSG1, KRT7, SLC7A2, RBM5) and functional verification of TNF-α/NF-κB pathway inhibition through techniques such as Western blotting, immunofluorescence, or siRNA-mediated knockdown will be important directions for follow-up work. Additionally, the current model employs an in vitro histamine induction approach; future studies employing in vivo keloid transplantation models would further substantiate the translational potential of HUCMSCs-Exos. These represent natural extensions of the current exploratory work that we intend to pursue in subsequent investigations.

## 5 Conclusions

Our experiment successfully establishes a histamine-induced keloid model in vitro. HUCMSCs-Exos effectively inhibit the proliferation of keloid fibroblasts, promoting apoptosis and exhibiting a dose-dependent effect. We also proved that HUCMSCs-Exos regulated genes like SLC7A2, RBM5, SLC25A5, and inhibit TNF-α/NF-κB signaling pathway by upregulating the expression of intracellular PSG1 and KRT7 gene, ultimately inhibiting keloid fibroblasts proliferation activity and enhancing apoptosis. These findings open new avenues for the use of MSC-Exos and suggest gene therapy as a potential treatment for patients with keloid.

## Statement

We have obtained informed consent from the donors for our experiments on human samples. We always respect the privacy rights of human subjects.

## Declaration of competing interest

All authors have no conflicts of interest to declare.

## Figures and Tables

**figs1:**
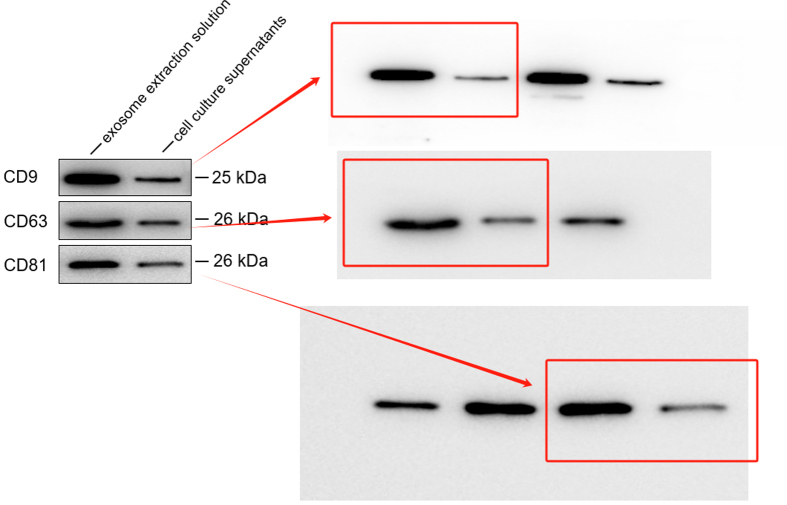


**figs2:**
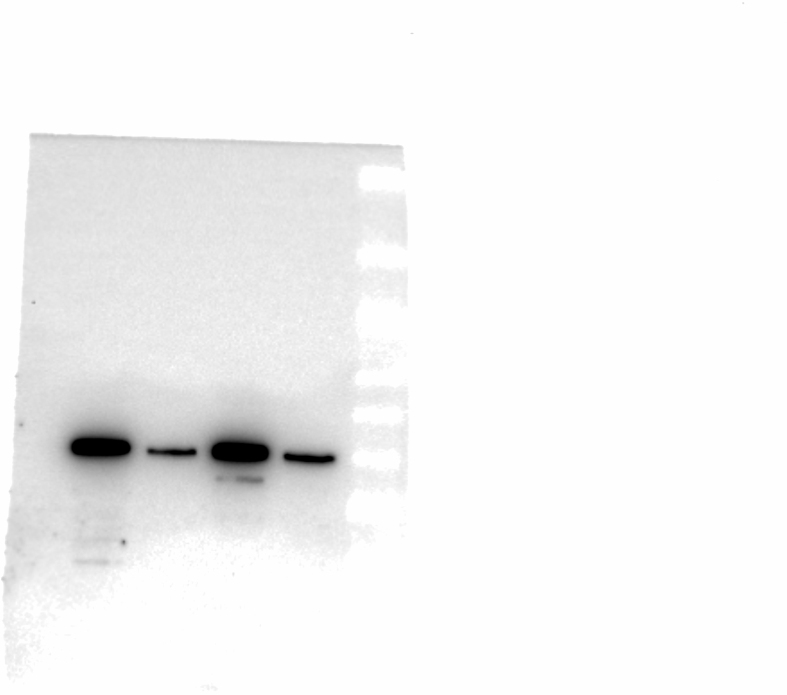


**figs3:**
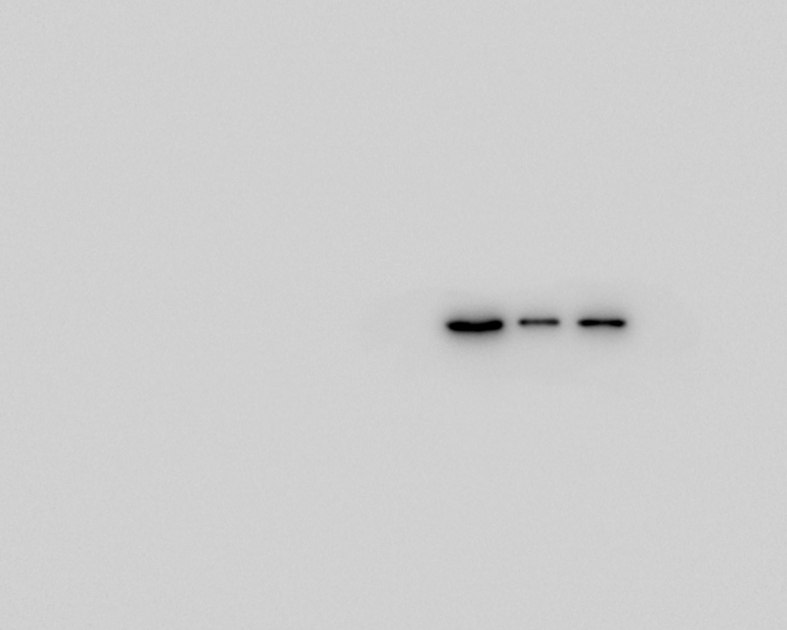


**figs4:**
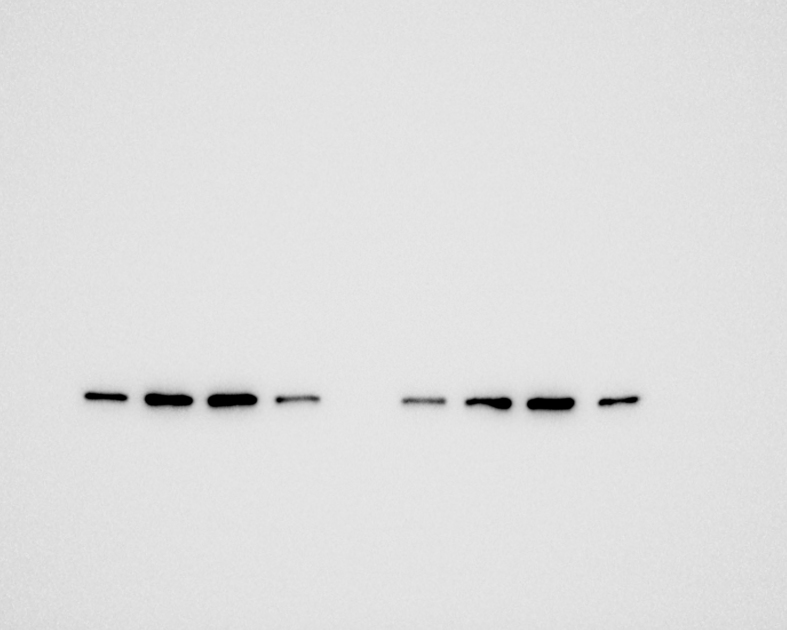


## Data Availability

Data will be made available on request.
